# Pathogenic Potential of Invasive Mucosa‐Associated *Klebsiella pneumoniae* Isolates in Ulcerative Colitis: Characterization of Virulence Factors and Inflammatory Response

**DOI:** 10.1002/iid3.70285

**Published:** 2025-10-15

**Authors:** Ensieh Olamafar, Abbas Yadegar, Shabnam Shahrokh, Maryam Tajabadi Ebrahimi, Hamidreza Houri

**Affiliations:** ^1^ Department of Biology Faculty of Sciences, Central Tehran Branch, Islamic Azad University Tehran Iran; ^2^ Foodborne and Waterborne Diseases Research Center, Research Institute for Gastroenterology and Liver Diseases Shahid Beheshti University of Medical Sciences Tehran Iran; ^3^ Gastroenterology and Liver Diseases Research Center Research Institute for Gastroenterology and Liver Diseases, Shahid Beheshti University of Medical Sciences Tehran Iran; ^4^ Celiac Disease and Gluten Related Disorders Research Center Research Institute for Gastroenterology and Liver Diseases, Shahid Beheshti University of Medical Sciences, Tehran, Iran

**Keywords:** inflammatory bowel diseases, interleukin‐8, *Klebsiella pneumoniae*, pathobionts, ulcerative colitis

## Abstract

**Background:**

*Klebsiella pneumoniae* has emerged as an opportunistic pathobiont potentially exacerbating ulcerative colitis (UC) through virulence‐mediated inflammation. However, the pathogenic role of invasive mucosa‐associated *K. pneumoniae* in UC remains underexplored. This study characterizes virulence factors and inflammatory responses in *K. pneumoniae* isolates from UC patients to elucidate their contribution to disease progression.

**Methods:**

Mucosa‐associated *K. pneumoniae* isolates (*n* = 62) from UC patients were compared to non‐UC controls. Virulence genes, including *kfu*, *magA*, *fimH*, *mrkD*, *rmpA*, *ybtS*, *iutA*, *iroN*, *hcp*, and *vgrG*, were investigated via PCR. Adhesion, invasion, and intracellular survival were assessed in Caco‐2 and THP‐1 cells. The relative expression levels of the *NOD1*, *NOD2*, *STAT1*, and *NF‐κB* genes were assessed using real‐time quantitative PCR. The concentrations of interleukin‐1β (IL‐1β), interleukin‐6 (IL‐6), and interleukin‐8 (IL‐8) were quantified with enzyme‐linked immunosorbent assay (ELISA) kits.

**Results:**

UC‐associated *K. pneumoniae* isolates exhibited higher prevalence of adhesion/invasion genes (e.g., *fimH* in 77.4% vs. 46.6%) and enhanced adhesive/invasive phenotypes (29% adherent, 16.1% invasive) compared to controls. These strains lacked hypervirulence markers (*rmpA*, *magA*) but aligned with classical virulent profiles. Invasive isolates upregulated *NOD1*, *NOD2*, and *STAT1* expression and significantly increased IL‐8 secretion (*p* < 0.05), amplifying pro‐inflammatory responses.

**Conclusion:**

Invasive mucosa‐associated *K. pneumoniae* in UC display distinct virulence traits that promote epithelial invasion and inflammation, potentially driving disease exacerbation. Targeting these pathobionts offers a promising avenue for novel UC therapies.

## Graphical abstract

This study was conducted in 83 patients with ulcerative colitis (UC). *Klebsiella pneumoniae* isolates showed a higher prevalence of virulence genes (*fimH*, *hcp*, *fyuA*, *irp1*) compared to non‐UC controls. UC isolates exhibited increased adhesion (29% vs. 8%) and invasion (16% vs. 3%), with invasive strains elevating *STAT1*, *NOD1*, *NOD2* expression, and IL‐8 production.

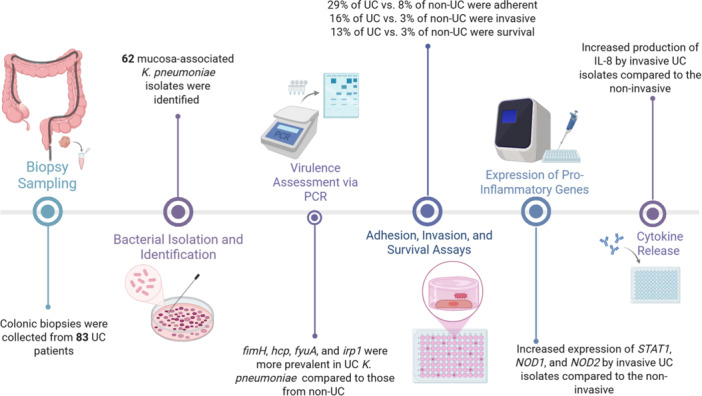

## 1 Introduction

Research on inflammatory bowel disease (IBD), which includes Crohn′s disease (CD) and ulcerative colitis (UC), has increasingly pointed to potential causes related to defects in host immune response and/or alterations in the microbial communities in the gut lumen [1, 2]. Any disruption of the normal balance between commensal and pathogenic bacteria, referred to as “dysbiosis”, may predispose individuals to IBD. Although dysbiosis has been recognized as a significant factor in the pathogenesis of IBD for nearly a decade, it remains uncertain whether it is a cause or a consequence of chronic intestinal inflammation. Additionally, it is unclear whether individuals transition from a healthy microbial state, known as “eubiosis”, to dysbiosis before developing IBD [3, 4].

The pursuit of identifying the bacterial species that initiate and sustain chronic inflammation in IBD remains ongoing. Earlier research focused on isolating a single pathogen responsible for triggering these immune responses. However, the current understanding of IBD pathogenesis has shifted to recognize that genetically or immunologically predisposed individuals may react abnormally to a complex dysbiotic gut environment, characterized by an overrepresentation of certain enteric bacteria. This imbalance leads to persistent immune activation and chronic inflammation. Notably, specific species within the Proteobacteria phylum, known as “pathobionts,” are central to this dysbiotic shift [5, 6, 7]. These pathobionts, including adherent‐invasive *Escherichia coli* (AIEC) [8, 9], *Bacteroides fragilis* [10], *Fusobacterium nucleatum* [11], and *Campylobacter concisus* [12] have been thoroughly studied in the context of IBD. These bacteria often exploit genetic vulnerabilities in pathogen recognition and bacterial clearance, disrupting the balance between harmful and protective gut microbiota. Additionally, several members of the Enterobacteriaceae family, such as *Klebsiella*, *Proteus*, *Yersinia*, and *Salmonella* species, have also been implicated in IBD [13, 14, 15]. Studies have consistently shown that these strains are more frequently isolated and detected in mucosal biopsies and resected specimens from IBD patients. They also exhibit intramucosal activity and pathogenicity and trigger elevated systemic antibody responses in IBD patients compared to controls.

Among these, *Klebsiella pneumoniae* has emerged as a significant pathobiont in IBD. In conjunction with the endogenous gut microbiota, *K. pneumoniae* has been shown to induce colitis in wild‐type mouse models [16]. Metagenomic and metabolomic analyses further reveal that *K. pneumoniae* is more prevalent in the colonic mucosa and fecal samples of IBD patients compared to healthy controls [17]. Our previous comprehensive metagenomic analysis of the Inflammatory Bowel Disease Multi'omics Database (IBDMDB) data sets established a strong link between *K. pneumoniae*‐related metabolic pathways and UC patients [18]. Recent research by Zhang et al. has identified a positive correlation between UC severity, as measured by the Mayo index, and the presence of *K. pneumoniae* [19]. Their findings demonstrated that *K. pneumoniae* isolated from the colon tissues of UC patients could colonize the colon, activate caspase‐11 inflammasomes, and contribute to intestinal inflammation. Despite these insights, there is still limited data on the pathogenic and inflammatory characteristics of mucosa‐associated *K. pneumoniae* isolated from colonic biopsies of UC patients. Therefore, this study aims to investigate the in vitro pathogenic potential of *K. pneumoniae* strains isolated from colonic biopsies of Iranian patients with UC.

## 2 Materials and Methods

### 2.1 Patients and Biopsy Samples

The study protocol was approved by the Institutional Ethical Review Committee of the Research Institute for Gastroenterology and Liver Diseases at Shahid Beheshti University of Medical Sciences. Written informed consent was obtained from all participants or their legal guardians before sample collection. A total of 83 patients diagnosed with UC were recruited from Taleghani Hospital in Tehran, Iran, between July 2022 and May 2024. UC diagnosis was confirmed through a combination of clinical evaluation, endoscopic findings, and histopathological analysis. During colonoscopy, biopsy samples were collected from inflamed regions of the colon using sterile forceps. Samples were immediately placed in brain heart infusion (BHI) broth (Merck, Germany) and transported to the microbiology laboratory at the Foodborne and Waterborne Diseases Research Center, Research Institute for Gastroenterology and Liver Diseases, Tehran, Iran, for further analysis. Furthermore, control samples were obtained from age‐ and sex‐matched individuals without IBD or other gastrointestinal pathologies. These individuals underwent colonoscopy as part of routine screening and exhibited no evidence of inflammatory, oncological, or other gastrointestinal conditions.

### 2.2 Bacterial Isolation and Identification

The fresh biopsy tissues were homogenized in BHI broth, inoculated onto MacConkey agar (Merck, Germany) plates, and incubated at 37°C for 24 h under aerobic conditions [20]. Single colonies displaying pink coloration on MacConkey agar, indicative of lactose fermentation, were selected for further analysis. *K. pneumoniae* strains were identified based on their morphological and biochemical properties, and molecular confirmation was performed using PCR amplification of the *khe* gene with specific primers (provided in Table S1) as previously described [21].

### 2.3 Virulence Detection of *K. pneumoniae* Isolates

Genomic DNA was extracted from mucosa‐associated *K. pneumoniae* isolates obtained from patients with IBD utilizing the QIAamp DNA Mini Kit (QIAGEN, Hilden, Germany), following the protocols provided by the manufacturer. The concentration of extracted DNA was measured using a NanoDrop ND‐1000 spectrophotometer, and its integrity was assessed by electrophoresis on a 0.8% agarose gel. DNA samples were stored at −20°C until further analysis by PCR. Virulence gene detection for colonic isolates of *K. pneumoniae* was conducted using PCR. Specific genes, including *kfu*, *magA*, *fimH*, *mrkD*, *rmpA*, *ybtS*, *iutA*, *iroN*, *hcp*, and *vgrG*, were targeted for identification. The PCR reaction was prepared using Taq DNA Polymerase Master Mix, specific primers (provided in Table S1), distilled water, and the DNA template. Amplification was conducted under the following conditions: initial denaturation at 95°C for 10 min, followed by 35 cycles of denaturation at 95°C for 30 s, annealing at 58°C for 45 s, and extension at 72°C for 45 s [22]. A final elongation step was carried out at 72°C for 10 min. The resultant PCR products were analyzed through gel electrophoresis for visualization.

### 2.4 Cell Lines and Culture Conditions

The human colorectal adenocarcinoma cell line, Caco‐2 (ATCC HTB‐37), was obtained from the Iranian Biological Resource Center in Tehran, Iran. Cells were cultured in high‐glucose Dulbecco's Modified Eagle Medium (H‐DMEM, Gibco, USA), supplemented with 1% penicillin (100 U/mL) and streptomycin (100 μg/mL), along with 10% heat‐inactivated fetal bovine serum (FBS, Gibco‐Invitrogen, Carlsbad, CA). Cultures were maintained in a humidified incubator with 5% CO2 at 37°C. Caco‐2 cells were seeded into 24‐well plates and grown to 80%–90% confluence under standard conditions. The medium was then replaced with serum‐free DMEM overnight. Cells were subsequently infected with *K. pneumoniae* isolates at a multiplicity of infection (MOI) of 10 for 24 h. Control groups consisted of untreated cells and cells treated with *E. coli* LF82 (as a confirmed adhesive/invasive strain) and DH5α (as a non‐pathogenic bacterium) under identical conditions for all identification processes, including adhesion, invasion, survival, and replication assays. For gene expression analysis and cytokine measurement, bacterial isolates were pretreated with polymyxin B to neutralize the effects of lipopolysaccharide (LPS) before incubation with Caco‐2 cells. All experiments were performed in duplicate and repeated a minimum of three times.

### 2.5 Cell Adhesion Assays

Adhesion, invasion, and survival assays were conducted according to the methodology established by Darfeuille‐Michaud et al. [23]. Accordingly, Caco‐2 cells were seeded in 24‐well plates at a density of 1 × 10^5^ cells per well and incubated in a 5% CO2 atmosphere at 37°C until they achieved 80%–90% confluence. Mid‐log‐phase bacteria *K. pneumoniae* isolates (2 × 10^6^; A600 = 0.4 to 0.6) were grown overnight in Luria‐Bertani (LB) broth (Merck, Germany), harvested during the exponential growth phase, and resuspended in antibiotic‐free DMEM. Caco‐2 monolayers were infected with bacteria at an MOI of 10 for 3 h at 37°C with 5% CO2. After infection, the monolayers were washed three times with PBS to remove nonadherent bacteria, lysed with 1% Triton X‐100 (Sigma‐Aldrich, UK), and serial dilutions were plated on LB agar (Merck, Germany) to determine the number of colony‐forming units (CFUs). An adhesion index of 1 or more, calculated as the average number of bacteria per cell, was considered indicative of positive adhesion. All assays were conducted in triplicate.

### 2.6 Cell Invasion Assays

The gentamicin protection assay was utilized to assess the invasive capacity of bacteria. Caco‐2 cells were seeded in 24‐well plates and grown to 80%–90% confluence at 37°C in 5% CO2. Confluent monolayers were infected with 10^6^ bacteria/mL at an MOI of 10 for 3 h. After the incubation period, cells were washed three times with PBS, and a fresh medium containing gentamicin (100 μg/mL, Sigma‐Aldrich, USA) was added to eliminate extracellular bacteria. The monolayers were incubated for an additional hour, then washed and lysed with 1% Triton X‐100. The number of intracellular bacteria was determined by plating on agar, and the invasion level was calculated as the percentage of the initial inoculum. Isolates with an invasion index of ≥ 0.1% were classified as invasive. All experiments were performed in triplicate [23].

### 2.7 Bacterial Survival and Replication Within Macrophages

The ability of bacteria to survive and replicate within macrophages was evaluated using a modified gentamicin protection assay [24]. THP‐1 cells were seeded into 24‐well plates at a density of 1 × 10^5^ cells per well and treated with 20 ng/mL PMA to induce differentiation into macrophages upon reaching 80%–90% confluence. Differentiated macrophages were infected with adherent/invasive *K. pneumoniae* isolates at an MOI of 10 and incubated at 37°C with 5% CO2 for 20 min. Following infection, monolayers were thoroughly washed with PBS, and fresh medium containing 100 μg/mL gentamicin was added to eliminate extracellular bacteria, marking 1‐h postinfection. Cells were then washed again and lysed with 1% Triton X‐100. To evaluate bacterial survival and replication 24 h postinfection, the medium was replaced with fresh medium containing 50 μg/mL gentamicin, and cells were incubated for an additional 23 h. All assays were conducted in triplicate. Isolates were defined as able to survive and replicate within macrophages, with survival ratios of 100% or more and replication ratios of 200% or more, respectively.

### 2.8 RNA Extraction and cDNA Synthesis

Total RNA was extracted from Caco‐2 cells using the RNeasy Plus Mini Kit (Qiagen, Germany), following the manufacturer's protocol. RNA concentration and purity were assessed using a NanoDrop spectrophotometer (ND‐1000, Thermo Scientific, USA) and confirmed by agarose gel electrophoresis. cDNA synthesis was performed using the BioFACT RT kit (BIOFACT, South Korea) as per the manufacturer′s instructions.

### 2.9 Gene Expression Analysis by RT‐qPCR

Gene expression levels were analyzed using real‐time quantitative PCR (RT‐qPCR) with the Rotor‐Gene Q instrument (Qiagen, Germany) and BioFACT 2× Real‐Time PCR Master Mix (BIOFACT, South Korea). Target genes and their corresponding primers used in the RT‐qPCR assays are listed in Table S1. The relative expression levels of *NOD1*, *NOD2*, *STAT1*, and *NF‐κB* were calculated using the $2^{‐∆∆C_{t}}$ method, with *GAPDH* serving as the internal control.

### 2.10 Measurement of Cytokines by ELISA

Following treatment, supernatants from the cell cultures were collected and stored at −20°C until analysis. Concentrations of interleukin‐1B (IL‐1B), interleukin‐6 (IL‐6), and interleukin‐8 (IL‐8) in the supernatants were quantified using enzyme‐linked immunosorbent assay (ELISA) kits (ZellBio GmbH, Germany), according to the manufacturer′s instructions.

### 2.11 Statistical Analysis

All statistical analyses were performed using R Statistical Software (version 4.0.5, R Foundation for Statistical Computing, Vienna, Austria) and GraphPad Prism (version 6.07, GraphPad Software, San Diego, CA). To assess differences in the frequency of virulence factors among groups, we employed Fisher′s exact and Chi‐squared tests, ensuring robust comparisons. For the gene expression data, we utilized the student's *t*‐test to evaluate differences between groups, followed by Tukey's post hoc test to account for multiple comparisons, thereby increasing the reliability of our results. The data presented in this study derive from three independent experiments, each comprising three biological replicates, allowing us to capture variability and enhance the statistical power. All results are reported as the mean ± standard error of the mean (SEM). We established a significance threshold at *p* < 0.05, with findings meeting this criterion classified as statistically significant. This rigorous approach facilitates a comprehensive understanding of the data and underscores the reliability of our conclusions.

## 3 Results

### 3.1 Bacterial Isolation and Virulence Determinants

We successfully isolated 62 mucosa‐associated strains of *K. pneumoniae* from individuals diagnosed with UC and utilized the same strains from a non‐UC cohort for comparative analysis. Colonoscopic and histopathological evaluations revealed that 54.8% (*n* = 34) of the *K. pneumoniae*‐positive UC patients presented with proctitis at the time of diagnosis. This was followed by left‐sided colitis in 19.3% (*n* = 12), backwash ileitis in 16.1% (*n* = 10), and pancolitis in 9.7% (*n* = 6). The UC cohort exhibited a median Mayo score of 8, with scores ranging from 5 to 12, indicating varying degrees of disease severity. Furthermore, PCR analyses identified the most prevalent virulence factors in the *K. pneumoniae* isolates from UC patients, including *ycfM*, which was detected in 100% (*n* = 62) of the strains, *hcp* in 93.5% (*n* = 58), and *mrkD* also in 93.5% (*n* = 58). Notably, our analysis revealed that the genes *fimH*, *hcp*, *fyuA*, and *irp1* were significantly more prevalent in UC‐associated *K. pneumoniae* compared to isolates from non‐UC individuals, suggesting a potential link between these virulence factors and the pathogenesis of UC. Based on the absence of hypervirulence markers such as *rmpA* and *magA*, and limited siderophore genes (*iutA* in 16.1%, *iroN* in 29%), these isolates align with classical virulent profiles rather than hypervirulent or ultravirulent strains. Table 1 provides a detailed overview of the prevalence of these virulence determinants in both the *K. pneumoniae* isolates from UC patients and those from the non‐UC cohort. This comprehensive analysis enhances our understanding of the role of mucosa‐associated *K. pneumoniae* in UC and highlights specific virulence factors that may contribute to disease manifestation.

**Table 1:** Distribution of virulence genes among mucosa‐associated *Klebsiella pneumoniae* isolated from patients with UC and non‐UC individuals.

Virulence determinants	UC‐associated *K. pneumonia* *n* = 31	Non‐UC‐associated *K. pneumoniae* *n* = 30	*p value*
Adhesins			
* mrkD*	29 (93.5%)	25 (83%)	0.25
* fimH*	24 (77.4%)	14 (46.6%)	0.02
* ycfM*	31 (100%)	28 (93.3%)	0.23
* kpn*	19 (61.3)	11 (36.7%)	0.09
Type VI Secretion System			
* hcp*	29 (93.5%)	11 (36.7%)	0.001
* vgrG*	13 (41.9%)	11 (36.7%)	0.87
Iron‐acquisition systems			
* ybtS*	11 (35.5%)	16 (53.3%)	0.25
* iutA*	5 (16.1%)	0	0.05
* iroN*	9 (29%)	2 (6.6%)	0.05
* fyuA*	23 (74.2%)	9 (30%)	0.001
* irp1*	25 (83%)	10 (33.3%)	0.001
Protectins			—
* magA*	0	0	—
* rmpA*	0	0	

### 3.2 Adhesion, Invasion, and Survival Ability of *K. pneumoniae*

**Figure 1:**
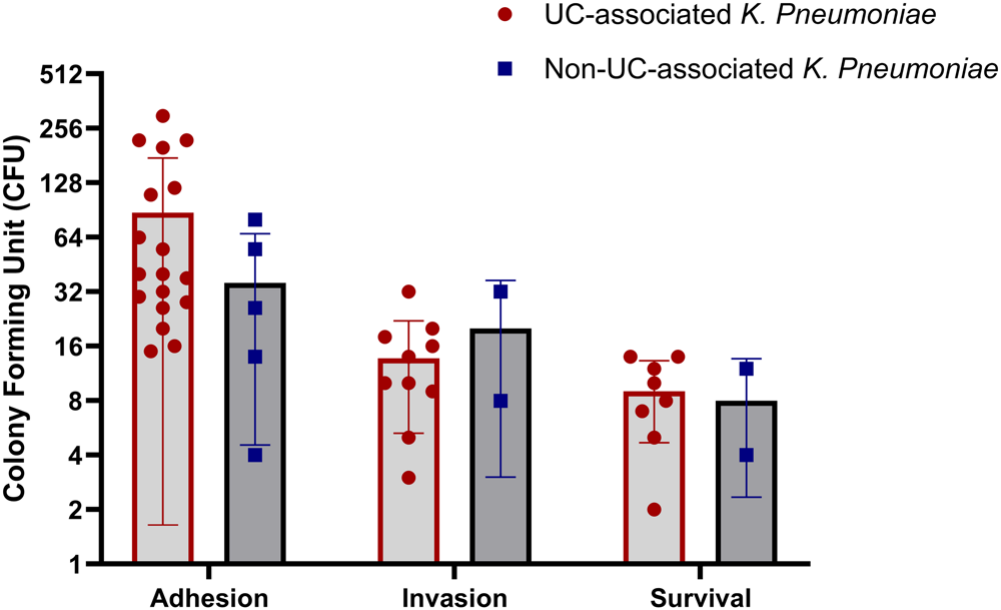
Adhesion and invasion capacity of *Klebsiella pneumoniae* isolates from UC‐associated and non‐UC‐associated sources. The panel shows the corresponding colony‐forming units (CFU) of adhered and intracellular bacteria. UC‐associated *K. pneumoniae* isolates demonstrated a higher propensity for adhesion and invasion compared to non‐UC‐associated strains, highlighting their potential role in mucosal disruption and persistence within intestinal epithelial cells.

The adhesion assay conducted on *K. pneumoniae* isolates in this study using Caco‐2 cells revealed that 18 isolates (29%) were categorized as adherent. In comparison, 8% (*n* = 5) of isolates recovered from non‐UC individuals displayed adhesive properties, indicating that adhesive isolates were significantly more prevalent among UC patients than non‐UC patients (*p* = 0.005). We further evaluated the invasiveness of the adherent *K. pneumoniae* isolates using a gentamicin protection assay. Among the 18 adhesive isolates, 10 (16.1%) were also found to be invasive; in contrast, only two non‐UC isolates demonstrated any invasive ability. To investigate the survival and replication capabilities within macrophages, we utilized the adhesive‐invasive isolates to infect THP‐1 cells. Accordingly, eight invasive *K. pneumoniae* isolates associated with UC and two non‐UC isolates exhibited the ability to survive and multiply inside THP‐1 cells. Table 2 and Figure 1 indicate the ability of *K. pneumoniae* isolates from UC and non‐UC cases in adhesion, invasion, and survival according to the number of isolates (Table 2) and colony counts (CFU) (Figure 1) after cell lysis using Triton X‐100.

**Table 2:** Prevalence of adhesion, invasion, and intracellular survival in *Klebsiella pneumoniae* isolates from UC patients and non‐UC individuals.

Characteristic	UC patients total isol ates: 62)	Non‐UC individuals Total Isolates: 62	Statistical significance
Adherent Isolates (Caco‐2 Cell Assay)	18 (29%)	5 (8%)	*p* = 0.005
Invasive Isolates (Gentamicin Protection Assay, Among Adherent Isolates)	10 (56% of adherent; 16.1% overall)	2 (40% of adherent; 3.2% overall)	*p* = 0.640
Isolates Surviving and Multiplying in Macrophages (THP‐1 Cell Infection, Among Invasive Isolates)	8 (80% of invasive)	2 (100% of invasive)	*p* = 1.000

### 3.3 Invasive UC‐Associated *K. pneumoniae* Isolates Upregulated STAT1, NOD1, and NOD2 Genes

**Figure 2:**
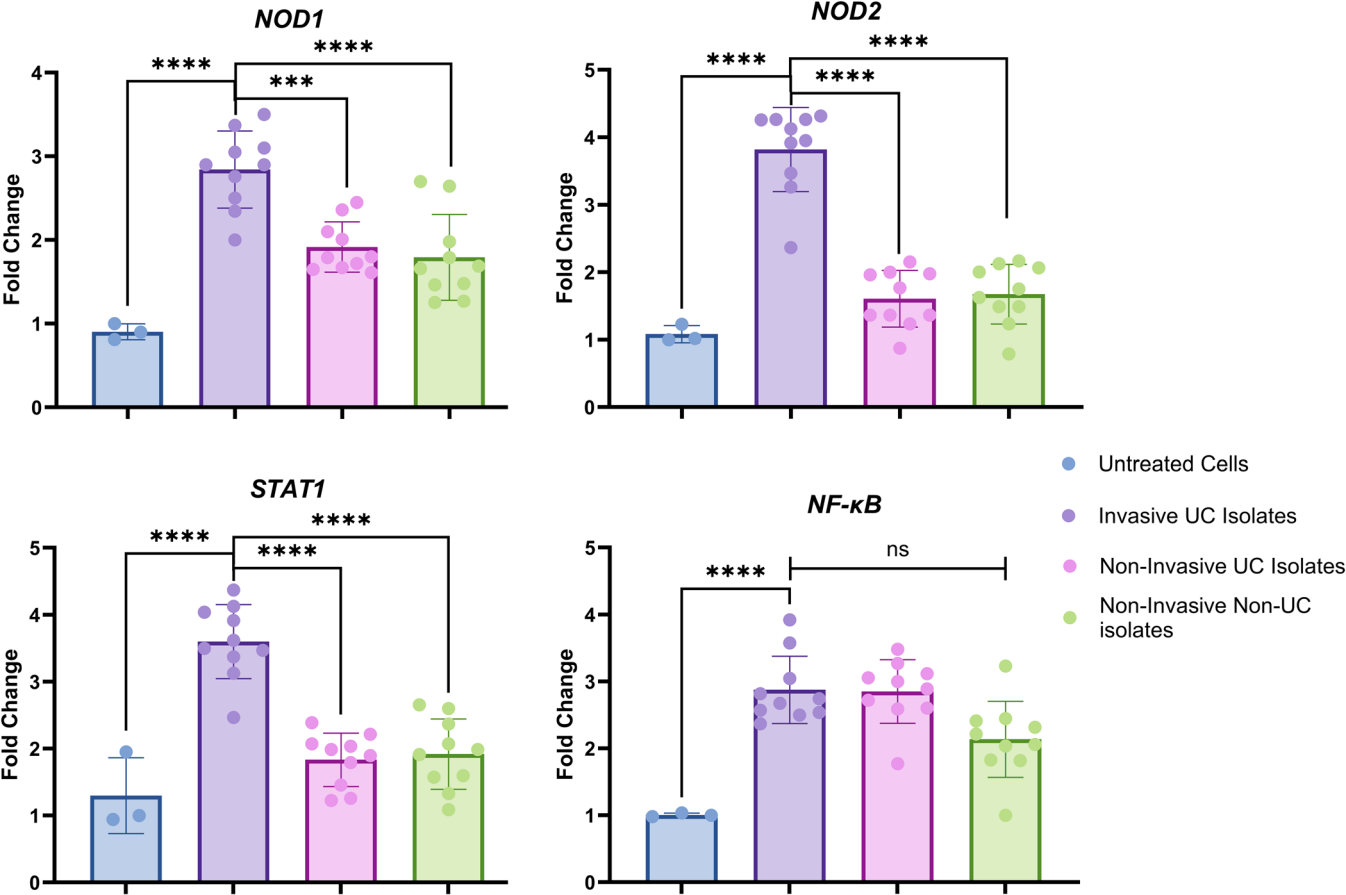
Relative expression levels of *NOD1*, *NOD2*, *STAT1*, and *NF‐κB* genes in Caco‐2 cells in response to invasive UC‐associated *K. pneumoniae* isolates. The expression levels are compared to those induced by noninvasive UC‐associated and non‐UC‐associated isolates. Results indicate a significant upregulation of the NOD1, NOD2, and STAT1 genes in Caco‐2 cells challenged with invasive isolates, suggesting a heightened immune response and potential involvement in the pathophysiology of UC. Statistical significance for comparisons with the invasive UC group is denoted as follows: ***p < 0.001 and ****p < 0.0001.

To investigate the ability of invasive UC‐associated *K. pneumoniae* isolates to alter activating immune response factors, we examine the expression of *STAT1*, *NOD1*, *NOD2*, and *NF‐κB* among UC‐associated *K. pneumoniae* isolates relative to noninvasive isolates. Importantly, we found markedly increased expression of *STAT1*, *NOD1*, and *NOD2*, but not *NF‐kB*, in Caco‐2 cells upon treatment with invasive UC‐associated isolates compared to the noninvasive UC‐associated as well as noninvasive non‐UC‐associated isolates (Figure 2). These findings indicated that the invasion of colonic cells by invasive strains could be a critical factor in the excessive expression of *STAT1* and *NOD1*, *NOD2*, which could be implicated in IBD pathogenesis.

### 3.4 Invasive UC‐Associated *K. pneumoniae* Isolates Promote Increased IL‐8 Release

**Figure 3:**
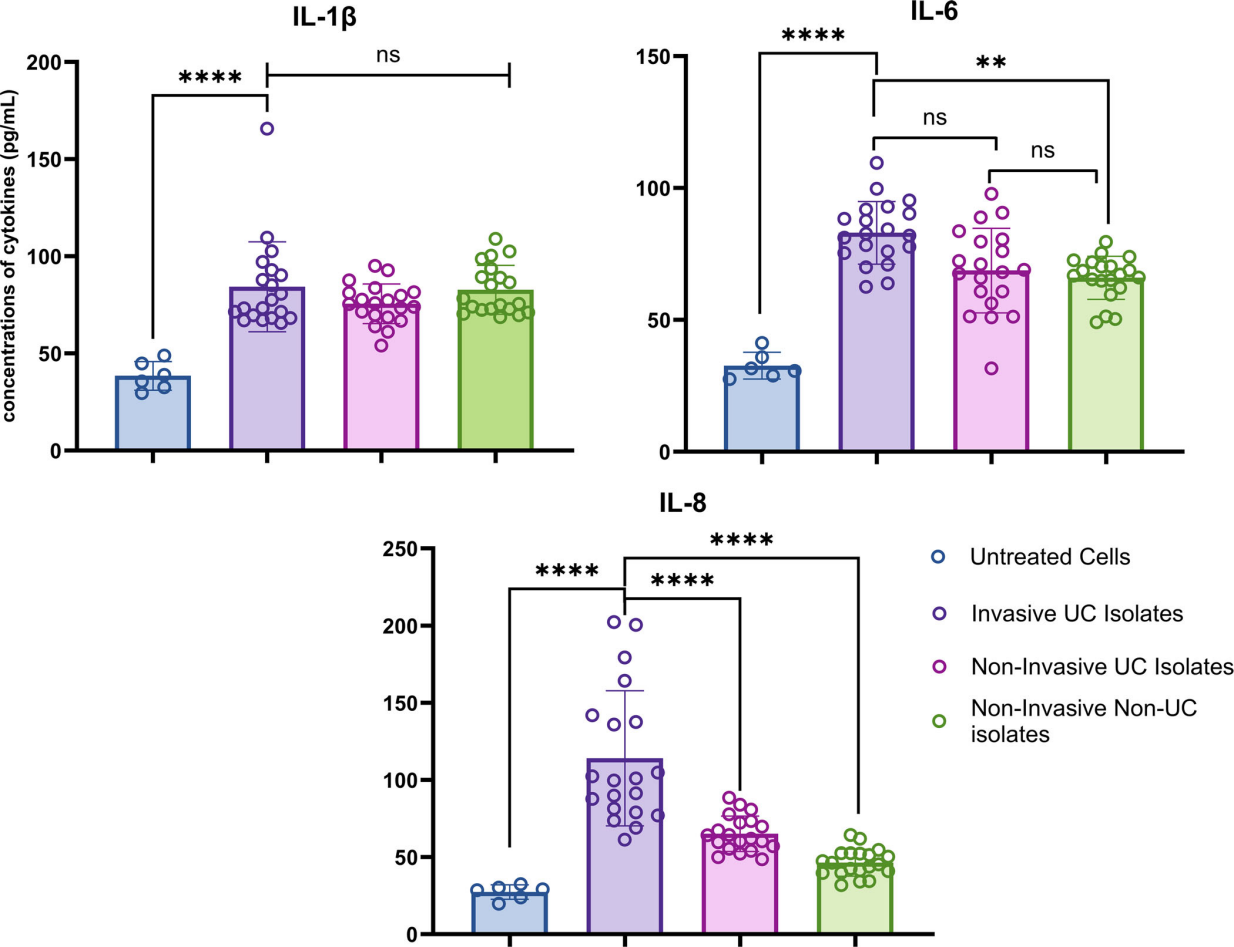
Release of IL‐1β, IL‐6, and IL‐8 in Caco‐2 cells induced by invasive UC‐associated *Klebsiella pneumoniae* isolates. The mean concentrations of cytokines (pg/mL) are presented, with error bars representing the standard deviation (SD) of triplicate measurements. Data illustrate the inflammatory response elicited by invasive isolates, highlighting their potential role in exacerbating mucosal inflammation in ulcerative colitis. Statistical significance for comparisons with the invasive UC group is denoted as follows: **p < 0.01 and ****p < 0.0001.

To evaluate the ability of UC‐associated *K. pneumoniae* isolates to induce the release of inflammatory cytokines, we measured the concentrations of IL‐1β, IL‐6, and IL‐8 using ELISA. The comparison was made between invasive UC‐associated *K. pneumoniae* isolates, noninvasive UC‐associated isolates, and noninvasive non‐UC‐associated isolates. Notably, invasive UC‐associated *K. pneumoniae* isolates significantly increased the production of IL‐8 compared to both noninvasive UC‐associated and noninvasive non‐UC‐associated isolates. Additionally, these invasive isolates also significantly elevated IL‐6 levels compared to noninvasive non‐UC‐associated isolates, though no significant difference was observed when compared to noninvasive UC‐associated isolates. There was no significant difference in IL‐1β levels among the tested groups. The concentrations of IL‐1β, IL‐6, and IL‐8 across these groups are illustrated in Figure 3.

## 4 Discussion

This study explored the virulence characteristics of mucosa‐associated *K. pneumoniae* isolates from UC patients, highlighting their potential role in UC pathogenesis. Our findings revealed that certain virulence factors, particularly those associated with adhesion, invasion, and immune modulation, were significantly more prevalent in *K. pneumoniae* strains from UC patients than those from non‐UC individuals. Importantly, isolates with invasive properties were linked to heightened immune responses, suggesting a key pathogen‐host interaction that may exacerbate inflammation and contribute to disease progression in UC. Our study reinforces previous research, demonstrating that the interactions between *K. pneumoniae*, intestinal epithelial cells, and the gut immune system play a pivotal role in the development of chronic intestinal inflammatory disorders, including IBD [16, 19, 25]. These findings provide valuable new insights into the contribution of this pathobiont to the persistence and exacerbation of gut inflammation, further supporting its potential role in disease progression.

Our study identified a significantly higher prevalence of the *fimH*, *hcp*, *fyuA*, and *irp1* virulence genes in mucosa‐associated *K. pneumoniae* isolates from UC patients compared to non‐UC controls. These genes, which are crucial for bacterial adhesion, invasion, and iron acquisition, are integral to bacterial colonization and persistence within the host environment. The elevated presence of these virulence factors suggests that UC‐associated *K. pneumoniae* may possess enhanced pathogenic capabilities relative to strains isolated from non‐UC individuals. This potential association between these specific virulence factors and UC pathogenesis implies their involvement in disease initiation and progression. Prior research has shown that such virulence factors contribute to gut inflammation and microbial dysbiosis, supporting our findings that these virulence determinants may promote bacterial persistence and invasion of colonic epithelial cells in UC patients. FimH, a type 1 fimbrial adhesin, has been shown to mediate the attachment of AIEC strains to carcinoembryonic antigen‐related cell adhesion molecule 6 (CEACAM6), which is abnormally expressed in the intestinal mucosa of a subset of IBD patients [26]. This interaction can further upregulate CEACAM6 expression through direct epithelial interaction and indirect TNF‐α secretion from infected macrophages, creating a feedback loop that promotes bacterial colonization and inflammation [26, 27]. In contrast, for *K. pneumoniae*, the MrkD adhesin of type 3 fimbriae is pivotal in mediating biofilm formation on extracellular matrix and collagen surfaces, promoting long‐term persistence in host environments such as the urinary tract and potentially the gut mucosa [28]. With *mrkD* detected in 93.5% of our isolates, this factor likely assumes greater significance than *fimH* in sustaining chronic inflammatory processes in UC.

Similarly, the type VI secretion system (T6SS), which includes the *hcp* gene, plays a crucial role in bacterial competition and enhances the fitness of *K. pneumoniae* within the intestinal microbiome [29, 30]. T6SS facilitates bacterial adhesion and invasion, promoting gut colonization and driving pro‐inflammatory immune responses. These mechanisms can disrupt microbial homeostasis, leading to a rise in pathobionts and exacerbating chronic inflammation, as seen in IBD [31]. Iron acquisition genes, such as *fyuA* and *irp1*, further enhance bacterial survival in the nutrient‐limited gut environment, potentially contributing to dysbiosis and immune dysregulation in genetically susceptible hosts [32, 33]. Given these findings, targeting key bacterial virulence factors, such as FimH and components of the T6SS, may offer novel therapeutic strategies to reduce inflammation and restore microbial balance in IBD. Recent studies suggest that inhibiting bacterial adhesion through FimH blockade can reduce mucosal inflammation in CD [34, 35], indicating that similar approaches could be applied to manage bacterial‐driven inflammation in UC. None of our isolates harbored the *rmpA* or *magA* genes, and only a limited subset carried siderophore‐related genes (*iutA* in 16.1% and *iroN* in 29%), indicating that these strains do not meet the criteria for hypervirulent or ultravirulent *K. pneumoniae*, as outlined in recent classifications of multidrug‐resistant strains exhibiting hypermucoviscous phenotypes [36, 37]. Instead, they conform to classical virulent phenotypes, demonstrating pronounced adhesion and invasion abilities despite the absence of extensive hypervirulence markers. However, it is important to note that *K. pneumoniae* strains can exhibit clinical aggressiveness even with a limited repertoire of virulence factors, as evidenced by studies demonstrating the coexistence of antibiotic resistance and virulence determinants in emerging clones, which may enhance pathogenicity through synergistic effects or resistance profiles [38, 39].

The adhesion and invasion assays further substantiated the pathogenic potential of UC‐associated *K. pneumoniae* isolates. Approximately 30% of isolates from UC patients demonstrated significant adhesion capabilities, compared to only ~8% from non‐UC individuals. Adhesion is a critical initial step in bacterial colonization, enabling the pathogen to establish itself within the mucosa, which can exacerbate colitis by promoting inflammation and compromising epithelial barriers, key processes in UC pathogenesis [6, 40]. Additionally, 16.1% of UC‐associated isolates exhibited invasive properties, allowing them not only to adhere to but also to invade Caco‐2 cells. Bacterial invasion of colonic epithelial cells may facilitate immune evasion, prolong infection, and amplify inflammatory responses. It is well‐established that pathobiont strains, upon invading intestinal epithelial cells, provoke a pro‐inflammatory environment [41]. This invasion is particularly relevant in chronic gastrointestinal inflammatory conditions like IBD [42]. The ability of these *K. pneumoniae* isolates to survive and replicate within macrophages further underscores their potential to persist and fuel chronic inflammation in UC.

Importantly, the study also demonstrated that invasive UC‐associated *K. pneumoniae* isolates elicited significant upregulation of *STAT1*, *NOD1*, and *NOD2* gene expression in Caco‐2 cells. These findings are particularly relevant given the established role of the NOD‐like receptor pathway in the recognition of bacterial pathogens and the activation of immune responses in IBD [43]. The upregulation of *NOD1* and *NOD2* in response to invasive strains suggests that these bacteria may trigger intracellular immune sensors, contributing to the exaggerated inflammatory response observed in UC. This aligns with prior studies demonstrating that dysregulation in *NOD2* is associated with increased susceptibility to IBD [44], further linking *K. pneumoniae* virulence to disease pathogenesis. Moreover, previous studies have demonstrated that certain enteropathogenic bacteria can activate the JAK/STAT pathway, which, in turn, stimulates β‐catenin signaling [45]. This activation promotes and sustains a pro‐carcinogenic inflammatory microenvironment in the colon. Such inflammatory modulators, when triggered in these contexts, may serve as potential biomarkers for assessing the risk and facilitating early detection of infection‐associated IBD. Future research should explore these pathways further to identify their role in the progression of IBD and their potential as therapeutic targets or diagnostic tools.

Our cytokine analysis revealed a marked increase in IL‐8 secretion in response to invasive *K. pneumoniae* isolates from UC patients, significantly higher compared to noninvasive UC‐associated and non‐UC‐associated isolates. IL‐8, a key pro‐inflammatory cytokine, plays a pivotal role in the pathogenesis of IBD by promoting neutrophil recruitment and amplifying inflammatory responses [46]. The elevated IL‐8 levels triggered by these invasive isolates likely intensify mucosal inflammation in colitis, thus contributing to the progression of UC. In addition, although IL‐6 levels were also increased in response to the invasive isolates, there was no significant difference in IL‐1β levels between the groups, indicating that *K. pneumoniae* selectively activates specific cytokine pathways. Recent evidence suggests that *K. pneumoniae* can also activate caspase‐11 in gut epithelial cells, leading to the release of interleukin‐18 (IL‐18), a cytokine with a key role in intestinal inflammation [19]. These interactions may further intensify the inflammatory processes characteristic of colitis and IBD, conditions in which elevated *K. pneumoniae* levels are frequently observed in affected individuals [18].

This study has several strengths and limitations that should be acknowledged. First, it provides a novel characterization of mucosa‐associated *K. pneumoniae* isolates from UC patients, focusing on their invasive potential and inflammatory effects in a region (Iran) underrepresented in IBD microbiome research, thereby contributing to global diversity in pathobiont studies. Second, the comprehensive approach, integrating virulence gene profiling, in vitro adhesion/invasion assays, macrophage survival tests, and quantification of immune responses (e.g., IL‐8 and gene expression, offers a multifaceted view of pathogenicity, bridging microbial traits with host inflammation. Third, the use of patient‐derived isolates and clinically relevant models (Caco‐2 and THP‐1 cells) enhances translational relevance, supporting the identification of *K. pneumoniae* as a therapeutic target in UC. Some limitations should be acknowledged in this study. First, virulence profiling was restricted to a selected panel of genes (e.g., *fimH*, *hcp*, *fyuA*, *irp1*), potentially overlooking other emerging factors or resistance determinants that could influence pathogenicity. Second, the absence of multilocus sequence typing (MLST) data hinders direct comparison of our isolates to established global clones, such as those associated with hypervirulence or antibiotic resistance in IBD contexts; future studies should incorporate MLST to explore associations between specific sequence types and invasive phenotypes. Additionally, the cross‐sectional design does not establish causality between *K. pneumoniae* invasion and UC progression, nor does it address longitudinal changes in microbial composition or inflammatory markers. Finally, whole genome sequencing of the isolates was not performed, which could provide further context on the clinical implications of these strains, particularly in therapeutic settings. These limitations highlight the need for larger, multicenter studies with in vivo models and comprehensive genomic analyses to validate and expand upon our observations.

In conclusion, our findings provide valuable insights into the potential involvement of invasive *K. pneumoniae* in UC pathogenesis, underscoring the relevance of virulence factors associated with adhesion, invasion, and immune modulation. The capacity of these pathobionts to survive intracellularly and evoke a robust immune response suggests that they may contribute to sustaining the prolonged, chronic inflammation characteristic of UC. These results also highlight *K. pneumoniae* pathobionts as a potential therapeutic target, particularly in UC patients harboring these pathogenic strains. Future research should aim to clarify the precise mechanisms through which these virulence factors modulate host immunity and promote chronic inflammation in UC. Understanding these pathways could unveil new therapeutic strategies for managing UC by targeting *K. pneumoniae*. Targeting these bacteria in therapeutic interventions could offer a promising approach for treating UC.

## Author Contributions

**Ensieh Olamafar:** methodology, investigation, visualization, writing – original draft. **Abbas Yadegar:** investigation and methodology. **Shabnam Shahrokh:** investigation, software, data curation, and formal analysis. **Maryam Tajabadi Ebrahimi:** methodology, formal analysis, software, resources, and data curation. **Hamidreza Houri:** conceptualization, funding acquisition, writing – review and editing, validation, software, project administration, supervision, data curation, and resources.

## Ethics Statement

This study was reviewed and approved by the Institutional Ethical Review Committee of the Research Institute for Gastroenterology and Liver Diseases at Shahid Beheshti University of Medical Sciences. The patients/participants provided their written informed consent to participate in this study.

## Consent

The authors have nothing to report.

## Conflicts of Interest

The authors declare no conflicts of interest.

## Supporting information


**Table S1:** Oligonucleotide printers used in this study.

## Data Availability

The data that supports the findings of this study are available in the supporting information of this article.

## References

[1] F. J. Ryan , A. M. Ahern , R. S. Fitzgerald , et al., “Colonic Microbiota Is Associated With Inflammation and Host Epigenomic Alterations in Inflammatory Bowel Disease,” Nature Communications 11, no. 1 (2020): 1512. 10.1038/s41467-020-15342-5 PMC7089947 32251296

[2] A. D. Kostic , R. J. Xavier , and D. Gevers , “The Microbiome in Inflammatory Bowel Disease: Current Status and the Future Ahead,” Gastroenterology 146, no. 6 (2014): 1489–1499. 24560869 10.1053/j.gastro.2014.02.009 PMC4034132

[3] D. Gevers , S. Kugathasan , D. Knights , A. D. Kostic , R. Knight , and R. J. Xavier , “A Microbiome Foundation for the Study of Crohn's Disease,” Cell Host & Microbe 21, no. 3 (2017): 301–304. 28279336 10.1016/j.chom.2017.02.012 PMC5684696

[4] K. Matsuoka and T. Kanai , “The Gut Microbiota and Inflammatory Bowel Disease,” Seminars in Immunopathology 37, no. 1 (2015): 47–55. 25420450 10.1007/s00281-014-0454-4 PMC4281375

[5] J. Chow , H. Tang , and S. K. Mazmanian , “Pathobionts of the Gastrointestinal Microbiota and Inflammatory Disease,” Current Opinion in Immunology 23, no. 4 (2011): 473–480. 21856139 10.1016/j.coi.2011.07.010 PMC3426444

[6] H. Yang , H. C. Mirsepasi‐Lauridsen , C. Struve , et al., “Ulcerative Colitis‐Associated *E. coli* Pathobionts Potentiate Colitis in Susceptible Hosts,” Gut Microbes 12, no. 1 (2020): 1847976. 33258388 10.1080/19490976.2020.1847976 PMC7781664

[7] A. Gilliland , J. J. Chan , T. J. De Wolfe , H. Yang , and B. A. Vallance , “Pathobionts in Inflammatory Bowel Disease: Origins, Underlying Mechanisms, and Implications for Clinical Care,” Gastroenterology 166, no. 1 (2024): 44–58. 37734419 10.1053/j.gastro.2023.09.019

[8] B. Nadalian , A. Yadegar , H. Houri , et al., “Prevalence of the Pathobiont Adherent‐Invasive *Escherichia coli* and Inflammatory Bowel Disease: A Systematic Review and Meta‐Analysis,” Journal of Gastroenterology and Hepatology 36, no. 4 (2021): 852–863. 32929762 10.1111/jgh.15260

[9] H. Kittana , J. C. Gomes‐Neto , K. Heck , et al., “Evidence for a Causal Role for *Escherichia coli* Strains Identified as Adherent‐Invasive (AIEC) in Intestinal Inflammation,” mSphere 8, no. 2 (2023): e0047822. 36883813 10.1128/msphere.00478-22 PMC10117065

[10] S. Rabizadeh , K. J. Rhee , S. Wu , et al., “Enterotoxigenic *Bacteroides fragilis*: A Potential Instigator of Colitis,” Inflammatory Bowel Diseases 13, no. 12 (2007): 1475–1483. 17886290 10.1002/ibd.20265 PMC3056612

[11] W. Su , Y. Chen , P. Cao , et al., “ *Fusobacterium nucleatum* Promotes the Development of Ulcerative Colitis by Inducing the Autophagic Cell Death of Intestinal Epithelial,” Frontiers in Cellular and Infection Microbiology 10 (2020): 594806. 33330137 10.3389/fcimb.2020.594806 PMC7728699

[12] K. F. Kirk , G. Méric , H. L. Nielsen , et al., “Molecular Epidemiology and Comparative Genomics of *Campylobacter concisus* Strains From Saliva, Faeces and Gut Mucosal Biopsies in Inflammatory Bowel Disease,” Scientific Reports 8, no. 1 (2018): 1902. 29382867 10.1038/s41598-018-20135-4 PMC5790007

[13] V. Baldelli , F. Scaldaferri , L. Putignani , and F. Del Chierico , “The Role of Enterobacteriaceae in Gut Microbiota Dysbiosis in Inflammatory Bowel Diseases,” Microorganisms 9, no. 4 (2021): 697. 33801755 10.3390/microorganisms9040697 PMC8066304

[14] C. Lupp , M. L. Robertson , M. E. Wickham , et al., “Host‐Mediated Inflammation Disrupts the Intestinal Microbiota and Promotes the Overgrowth of Enterobacteriaceae,” Cell Host & Microbe 2, no. 2 (2007): 119–129. 18005726 10.1016/j.chom.2007.06.010

[15] B. Willing , J. Halfvarson , J. Dicksved , et al., “Twin Studies Reveal Specific Imbalances in the Mucosa‐Associated Microbiota of Patients With Ileal Crohn's Disease,” Inflammatory Bowel Diseases 15, no. 5 (2009): 653–660. 19023901 10.1002/ibd.20783

[16] S. Federici , S. Kredo‐Russo , R. Valdés‐Mas , et al., “Targeted Suppression of Human IBD‐Associated Gut Microbiota Commensals by Phage Consortia for Treatment of Intestinal Inflammation,” Cell 185, no. 16 (2022): 2879–2898.e24. 35931020 10.1016/j.cell.2022.07.003

[17] X. Xu , D. K. W. Ocansey , S. Hang , et al., “The Gut Metagenomics and Metabolomics Signature in Patients With Inflammatory Bowel Disease,” Gut Pathogens 14, no. 1 (2022): 26. 35729658 10.1186/s13099-022-00499-9 PMC9215062

[18] B. Khorsand , H. Asadzadeh Aghdaei , E. Nazemalhosseini‐Mojarad , B. Nadalian , B. Nadalian , and H. Houri , “Overrepresentation of Enterobacteriaceae and *Escherichia coli* Is the Major Gut Microbiome Signature in Crohn's Disease and Ulcerative Colitis; a Comprehensive Metagenomic Analysis of IBDMDB Datasets,” Frontiers in Cellular and Infection Microbiology 12 (2022): 1015890. 36268225 10.3389/fcimb.2022.1015890 PMC9577114

[19] Q. Zhang , X. Su , C. Zhang , et al., “ *Klebsiella pneumoniae* Induces Inflammatory Bowel Disease Through Caspase‐11‐Mediated IL18 in the Gut Epithelial Cells,” Cellular and Molecular Gastroenterology and Hepatology 15, no. 3 (2023): 613–632. 36436756 10.1016/j.jcmgh.2022.11.005 PMC9871440

[20] B. Nadalian , B. Nadalian , H. Houri , et al., “Phylogrouping and Characterization of *Escherichia coli* Isolated From Colonic Biopsies and Fecal Samples of Patients With Flare of Inflammatory Bowel Disease in Iran,” Frontiers in Medicine 9 (2022): 985300. 36106322 10.3389/fmed.2022.985300 PMC9464868

[21] P. Li , D. Zhang , H. Li , J. Pang , H. Guo , and J. Qiu , “Establishment and Application of Multiplex PCR for Simultaneously Detecting *Escherichia coli*, *Salmonella*, *Klebsiella pneumoniae*, and *Staphylococcus aureus* in Minks,” Frontiers in Veterinary Science 7 (2020): 588173. 33313077 10.3389/fvets.2020.588173 PMC7704438

[22] M. Zhou , Y. Lan , S. Wang , et al., “Epidemiology and Molecular Characteristics of the Type VI Secretion System in *Klebsiella pneumoniae* Isolated From Bloodstream Infections,” Journal of Clinical Laboratory Analysis 34, no. 11 (2020): e23459. 32656871 10.1002/jcla.23459 PMC7676210

[23] A. Darfeuille‐Michaud , J. Boudeau , P. Bulois , et al., “High Prevalence of Adherent‐Invasive *Escherichia coli* Associated With Ileal Mucosa in Crohn's Disease,” Gastroenterology 127, no. 2 (2004): 412–421. 15300573 10.1053/j.gastro.2004.04.061

[24] A. L. Glasser , J. Boudeau , N. Barnich , M. H. Perruchot , J. F. Colombel , and A. Darfeuille‐Michaud , “Adherent Invasive *Escherichia coli* Strains From Patients With Crohn's Disease Survive and Replicate Within Macrophages Without Inducing Host Cell Death,” Infection and Immunity 69, no. 9 (2001): 5529–5537. 11500426 10.1128/IAI.69.9.5529-5537.2001 PMC98666

[25] S. Kitamoto , H. Nagao‐Kitamoto , Y. Jiao , et al., “The Intermucosal Connection Between the Mouth and Gut in Commensal Pathobiont‐Driven Colitis,” Cell 182, no. 2 (2020): 447–462.e14. 32758418 10.1016/j.cell.2020.05.048 PMC7414097

[26] N. Barnich , F. A. Carvalho , A. L. Glasser , et al., “CEACAM6 Acts as a Receptor for Adherent‐Invasive *E. coli*, Supporting Ileal Mucosa Colonization in Crohn Disease,” Journal of Clinical Investigation 117, no. 6 (2007): 1566–1574. 17525800 10.1172/JCI30504 PMC1868786

[27] N. Dreux , J. Denizot , M. Martinez‐Medina , et al., “Point Mutations in Fimh Adhesin of Crohn's Disease‐Associated Adherent‐Invasive *Escherichia coli* Enhance Intestinal Inflammatory Response,” PLoS Pathogens 9, no. 1 (2013): e1003141. 23358328 10.1371/journal.ppat.1003141 PMC3554634

[28] C. Vuotto , F. Longo , C. Pascolini , et al., “Biofilm Formation and Antibiotic Resistance in *Klebsiella pneumoniae* Urinary Strains,” Journal of Applied Microbiology 123, no. 4 (2017): 1003–1018. 28731269 10.1111/jam.13533

[29] P. F. Hsieh , Y. R. Lu , T. L. Lin , L. Y. Lai , and J. T. Wang , “ *Klebsiella pneumoniae* Type VI Secretion System Contributes to Bacterial Competition, Cell Invasion, Type‐1 Fimbriae Expression, and In Vivo Colonization,” Journal of Infectious Diseases 219, no. 4 (2019): 637–647. 30202982 10.1093/infdis/jiy534 PMC6350951

[30] T. Merciecca , S. Bornes , L. Nakusi , et al., “Role of *Klebsiella pneumoniae* Type VI Secretion System (T6SS) in Long‐Term Gastrointestinal Colonization,” Scientific Reports 12, no. 1 (2022): 16968. 36216848 10.1038/s41598-022-21396-w PMC9550808

[31] L. P. Allsopp , P. Bernal , L. M. Nolan , and A. Filloux , “Causalities of War: The Connection Between Type VI Secretion System and Microbiota,” Cellular Microbiology 22, no. 3 (2020): e13153. 31872954 10.1111/cmi.13153 PMC7540082

[32] K. A. Abdelhalim , A. Uzel , and N. Gülşen Ünal , “The Role of Major Virulence Factors and Pathogenicity of Adherent‐Invasive *Escherichia coli* in Patients With Crohn's Disease,” Gastroenterology Review 15, no. 4 (2020): 279–288. 33777266 10.5114/pg.2020.93235 PMC7988836

[33] L. Spiga , R. T. Fansler , Y. R. Perera , et al., “Iron Acquisition by a Commensal Bacterium Modifies Host Nutritional Immunity During Salmonella Infection,” Cell Host & Microbe 31, no. 10 (2023): 1639–1654. 37776864 10.1016/j.chom.2023.08.018 PMC10599249

[34] G. Chevalier , A. Laveissière , G. Desachy , et al., “Blockage of Bacterial FimH Prevents Mucosal Inflammation Associated With Crohn's Disease,” Microbiome 9, no. 1 (2021): 176. 34425887 10.1186/s40168-021-01135-5 PMC8383459

[35] A. Sivignon , J. Bouckaert , J. Bernard , S. G. Gouin , and N. Barnich , “The Potential of FimH as a Novel Therapeutic Target for the Treatment of Crohn′S Disease,” Expert Opinion on Therapeutic Targets 21, no. 9 (2017): 837–847. 28762293 10.1080/14728222.2017.1363184

[36] N. Tang , Y. Li , S. Yao , et al., “Epidemicity and Clonal Replacement of Hypervirulent Carbapenem‐Resistant *Klebsiella pneumoniae* With Diverse Pathotypes and Resistance Profiles in a Hospital,” Journal of Global Antimicrobial Resistance 32 (2023): 4–10. 36400407 10.1016/j.jgar.2022.11.007

[37] B. Douradinha , “Should Multidrug Resistant *Klebsiella pneumoniae* Strains Displaying Hypervirulent Traits Be Reclassified as Either Ultravirulent or Supervirulent?,” Microbiological Research 275 (2023): 127446. 37422962 10.1016/j.micres.2023.127446

[38] G. Di Mento , F. Gona , G. Russelli , et al., “A Retrospective Molecular Epidemiological Scenario of Carbapenemase‐Producing *Klebsiella pneumoniae* Clinical Isolates in a Sicilian Transplantation Hospital Shows a Swift Polyclonal Divergence Among Sequence Types, Resistome and Virulome,” Microbiological Research 256 (2022): 126959. 34995971 10.1016/j.micres.2021.126959

[39] D. D'Apolito , F. Arena , V. Conte , et al., “Phenotypical and Molecular Assessment of the Virulence Potential of KPC‐3‐producing *Klebsiella pneumoniae* ST392 Clinical Isolates,” Microbiological Research 240 (2020): 126551. 32652494 10.1016/j.micres.2020.126551

[40] L. C.‐H. Yu , “Microbiota Dysbiosis and Barrier Dysfunction in Inflammatory Bowel Disease and Colorectal Cancers: Exploring a Common Ground Hypothesis,” Journal of Biomedical Science 25, no. 1 (2018): 79. 30413188 10.1186/s12929-018-0483-8 PMC6234774

[41] S. Subramanian , J. M. Rhodes , A. C. Hart , et al., “Characterization of Epithelial IL‐8 Response to Inflammatory Bowel Disease Mucosal *E. coli* and Its Inhibition by Mesalamine,” Inflammatory Bowel Diseases 14, no. 2 (2008): 162–175. 17941093 10.1002/ibd.20296 PMC7108638

[42] E. Read , M. A. Curtis , and J. F. Neves , “The Role of Oral Bacteria in Inflammatory Bowel Disease,” Nature Reviews Gastroenterology & Hepatology 18, no. 10 (2021): 731–742. 34400822 10.1038/s41575-021-00488-4

[43] S. J. Rubino , T. Selvanantham , S. E. Girardin , and D. J. Philpott , “Nod‐Like Receptors in the Control of Intestinal Inflammation,” Current Opinion in Immunology 24, no. 4 (2012): 398–404. 22677577 10.1016/j.coi.2012.04.010

[44] Y. Zhou , S. Yu , and W. Zhang , “NOD‐Like Receptor Signaling Pathway in Gastrointestinal Inflammatory Diseases and Cancers,” International Journal of Molecular Sciences 24, no. 19 (2023): 14511. 37833958 10.3390/ijms241914511 PMC10572711

[45] R. Lu , Y. Zhang , and J. Sun , “STAT3 Activation in Infection and Infection‐Associated Cancer,” Molecular and Cellular Endocrinology 451 (2017): 80–87. 28223148 10.1016/j.mce.2017.02.023 PMC5469714

[46] J. Zhao , Q. Lu , Y. Liu , et al., “Th17 Cells in Inflammatory Bowel Disease: Cytokines, Plasticity, and Therapies,” Journal of Immunology Research 2021 (2021): 1–14. 10.1155/2021/8816041 PMC7846404 33553436

