# Supplementary material for: Pathogenic Potential of Invasive Mucosa‐Associated Klebsiella pneumoniae Isolates in Ulcerative Colitis: Characterization of Virulence Factors and Inflammatory Response
Source: Immun Inflamm Dis. 2025 Oct 15;13(10):e70285. doi: 10.1002/iid3.70285 (PMC12521864; doi:10.1002/iid3.70285)
Supplement: Supplementary file 1 — Table S1: Oligonucleotide printers used in this study. [file IID3-13-e70285-s001.pdf]

Pathogenic Potential of Invasive Mucosa-Associated *Klebsiella pneumoniae* Isolates in Ulcerative Colitis: Characterization of Virulence Factors and Inflammatory Response

Table S1. Oligonucleotide printers used in this study.

Targeted Gene	Function	Sequence	Tm (°C)	Product Size (bp)	Reference
khe	Encodes a hemolysin that facilitates tissue damage and nutrient acquisition by lysing host cells, contributing to the virulence of *Klebsiella pneumoniae*.	F: CGATGCTACTTATCCCGACA<br>R: ACCACCAGCAGACGAACTT	58<br>59	303	Li et al. (2020)
Capsular type K1	Encodes the serotype K1-specific capsular polysaccharide polymerase, responsible for capsule biosynthesis, which confers mucoviscosity, resistance to phagocytosis, and enhanced virulence in invasive infections such as liver abscesses.	F: GGTGCTCTTTACATCATTGC<br>R: GCAATGGCCATTTGCGTTAG	59.7<br>67.2	1283	Fang et al. (2004)
Capsular type K2	Encodes a serotype K2-specific gene in the capsular polysaccharide synthesis locus, involved in capsule production that enhances resistance to host immune responses and contributes to hypervirulence in disseminated infections.	F: GACCCGATATTCATACTTGACAGAG<br>R: CCTGAAGTAAAATCGTAAATAGATGGC	63.5<br>64.3	641	Turton et al. (2008)
Capsular type K5	Encodes a serotype K5-specific gene in the capsular polysaccharide synthesis locus, facilitating capsule formation that protects against complement-mediated killing and phagocytosis, thereby promoting bacterial persistence.	F: TGGTAGTGATGCTCGCGA<br>R: CCTGAACCCACCCCAATC	64.4<br>65.2	280	Turton et al. (2008)
Capsular type K54	Encodes a serotype K54-specific gene in the capsular polysaccharide synthesis locus, involved in capsule biosynthesis that enhances evasion of host immunity and is associated with invasive infections.	F: CATTAGCTCAGTGGTTGGCT<br>R: GCTTGACAAACACCATAGCAG	61.8<br>62.2	881	Fang et al. (2007)
Capsulartype K57	Encodes the serotype K57-specific capsular polysaccharide polymerase (wzy_K57), essential for capsule synthesis, which provides protection against host defenses and is linked to pyogenic liver abscess.	F: CTCAGGGCTAGAAGTGTCAT<br>R: CACTAACCCAGAAAGTCGAG	58.5<br>59.0	1037	Fang et al. (2007)
Capsulartype K20	Encodes a serotype K20-specific gene in the capsular polysaccharide synthesis locus, contributing to capsule production that aids in immune evasion and is prevalent in strains causing severe infections.	F: CGGTGCTACAGTGCATCATT<br>GTTATACGATGCTCAGTCGC	63.7<br>59.9	741	Fang et al. (2007)

mrkD	Encodes the adhesin subunit of type 3 fimbriae, facilitating bacterial adherence to extracellular matrix components such as collagen, promoting biofilm formation and persistence on biotic and abiotic surfaces.	F: AAGCTATCGCTGTACTTCCGGCA<br>R: GGCGTTGGCGCTCAGATAGG	64.16<br>63.58	340	Mao Zhou (2020)
Kfu	Encodes an iron uptake system involved in ferric iron transport, enhancing bacterial survival and proliferation in iron-limited host environments during infection.	F: GGCCTTTGTCCAGAGCTACG<br>GGGTCTGGCGCAGAGTATGC	60.74<br>63.26	638	Mao Zhou (2020)
ybtS	Encodes an enzyme involved in the biosynthesis of yersiniabactin, a siderophore that scavenges iron from the host, supporting bacterial growth and virulence during infection.	F: GACGGAAACAGCACGGTAAA<br>R: GAGCATAATAAGGCGAAAGA	58.78<br>53.2	242	Mao Zhou (2020)
iucA	Encodes aerobactin synthetase IucA, a nonribosomal peptide synthetase-independent siderophore synthetase involved in aerobactin biosynthesis for iron acquisition, contributing to hypervirulence.	F: GCATAGGCGGATACGAACAT<br>R: CACAGGGCAATTGCTTACCT	57.92<br>58.5	556	Mao Zhou (2020)
iroB	Encodes a glycosyltransferase that modifies enterobactin to salmochelin, a siderophore enabling iron scavenging while evading host lipocalin-2 sequestration, enhancing pathogenicity.	F: TGTGTGCTGTGGGTGAAAGC<br>R: ATGTTCGGTGAGATTCGCCAGT	61.38<br>62.31	2711	Mao Zhou (2020)
entB	Encodes a bifunctional isochorismatase/aryl carrier protein involved in enterobactin siderophore biosynthesis, facilitating iron acquisition essential for bacterial survival in the host.	F: GTCAACTGGGCCTTTGAGCCGTC<br>R: TATGGGCGTAAACGCCGGTGAT	66.13<br>64.63	400	Mao Zhou (2020)
hcp	Encodes hemolysin-coregulated protein, a core component of the type VI secretion system (T6SS), involved in interbacterial competition and delivery of effectors to host cells.	F: TCCCGACCGATAACAACACC<br>R: GATGTCGTGCATCAGGGGAT	59.75<br>59.89	242	Mao Zhou (2020)
vgrG	Encodes valine-glycine repeat G protein, a structural and effector component of the T6SS, facilitating bacterial competition and potentially contributing to host cell invasion and virulence.	F: TGAGCGTGTTTGTGCGAAAG<br>R: TGACGCCCGTAATATCCTGC	59.97<br>59.97	259	Mao Zhou (2020)
STAT	Encodes signal transducer and activator of transcription 1 (STAT1), a transcription factor in interferon signaling that coordinates epithelial cell responses, promotes autophagy, antimicrobial peptide production, and maintains intestinal barrier integrity during bacterial infections.	F: TGGCCCCTTGGATTGAGAGT<br>R: ATTGGCTTCTCAAGATACCTGCT	60<br>61	196	This study

NOD1	Encodes nucleotide-binding oligomerization domain-containing protein 1, an intracellular pattern recognition receptor that detects peptidoglycan fragments from Gram-negative bacteria, activating NF-κB and MAPK pathways to initiate inflammatory and innate immune responses in intestinal epithelial cells.	F: ACGATGAAGTGGCAGAGAGTT<br><br>R: GGCAGTCCCCTTAGCTGTGA	59<br><br>63	101	This study
NOD2	Encodes nucleotide-binding oligomerization domain-containing protein 2, a cytosolic sensor for muramyl dipeptide in bacterial cell walls, promoting NF-κB activation, autophagy, bacterial clearance, and protection against colitis through modulation of inflammatory pathways in intestinal epithelial cells.	F: GGGGTTTCGTCAGCCAGTAT<br><br>R: GAAGGAAGGCAGCCAATCCA	60<br><br>60	123	This study
NFKB	Encodes nuclear factor kappa B, a central transcription factor complex that regulates genes involved in inflammation, immune activation, and cell survival; activated downstream of receptors like NOD1 and NOD2 in response to bacterial pathogens, driving pro-inflammatory cytokine expression in intestinal epithelial cells.	F: GGATTTCGTTTCCGTTATGTATG<br><br>R: TCCTTGGGTCCAGCAGTTA	59<br><br>57	233	This study
NLRP3	Encodes NLR family pyrin domain-containing 3, a component of the NLRP3 inflammasome that senses microbial components and cellular damage, leading to caspase-1 activation, secretion of IL-1β and IL-18, and pyroptosis, thereby regulating intestinal inflammation, limiting pathogen colonization, and maintaining epithelial barrier integrity during bacterial infections.	F: GATCTTCGCTGCGATCAACA<br><br>R: GGGATTCGAAACACGTGCATTA	58<br><br>60	94	This study
